# A Simple Method of Swaging Tin Plates, for Vulcanite Work, without a Swager

**Published:** 1899-02

**Authors:** Vernon Knowles

**Affiliations:** L.D.S. (Eng.)


### Abstracts and Translations.

### A SIMPLE METHOD OF SWAGING TIN PLATES, FOR VULCANITE WORK, WITHOUT A SWAGER.[1]

BY VERNON KNOWLES, L.D.S. (ENG.).

PROSTHETIC dentistry being one of the most important branches of our profession, I feel I need hardly apologize for bringing the subject of my paper and demonstration before your notice, seeing that it treats in a practical way a subject that is of vital importance to us all. The days for making trial plates with wax palates are nearly if not quite over, and ere long any practitioner who continues to do so will, indeed, be a *rara avis*.

The advantages of using soft metal (tin) as a base-plate are so obvious that I need only just mention a few of the most important in passing. Perhaps the first and chief advantage is that we get our vulcanite plates of *even thickness all through;* hence they are much stronger, and the plates can be made thinner and therefore lighter, also the rugæ come out well marked. Then, again, soft metal base-plates are practically ridged, hence there is no risk of getting a false bite; and from the patient's point of view they are much more comfortable. It would perhaps be as well, before bringing before your notice the method that I have been using for some time past, to take a glance at the swagers in use at the present

[1] Read at the annual meeting, held at Bath, May, 1898.

moment. If my memory serves me right, the first that came out was Mr. Humby's. This swager, as most of you are aware, is practically a miniature vulcanizer, and by means of the steam generated the soft metal (meta-metal) is "blown" on to the plaster model. The essential defect in this method is, that, as the meta-metal has to be clamped down tight to prevent the steam escaping, the portion that is forced onto the model naturally becomes stretched, so that the metal over the middle of the palate is much thinner than at the sides, and in the case of deep palates this often occurs to such an extent that the steam forces its way right through the metal, when of course it is impossible to get the plate home. Then again, the steam has to be let out quickly, each time a plate is blown up, to prevent a vacuum being formed, and as three plates are usually required, this process becomes very tedious, not to say dirty. I may say that I have it on the authority of Mr. Humby himself, that his invention was not in the first place intended for this purpose, but for quite a different one, and was afterwards adapted for use in the dental laboratory. Nevertheless, I think you will all agree that, in spite of its apparent defects, Mr. Humby's swager was a step—in fact, the first step—in the right direction.

The next, I believe, was Mr. Gartrell's swager, which, whilst being a distinct improvement on the one already alluded to, is by no means perfect, as any one who has used it must admit. The thread on the screw, for some reason or another, is always getting out of order, and, a more important objection still, the shot have a way of losing their rotundity, and thus their use. Then, of course, remains the fact that one must have two impressions to work to, one for casting in fusible metal for swaging the plates, and the other for vulcanizing on, which entails more work and loss of time.

The most satisfactory swager I have kept until last, and that is Grundy's. This swager, as far as I know, and I have gone carefully into the matter, really does all that its inventor claims for it, a fact that I am sorry to say is all too rare, for even in dental appliances, as a modern bard has it, "things are not what they seem." But doubtless it has occurred to you that this swager, with all its advantages, is, at the price of five pounds, really dear, seeing that all it accomplishes is nothing more nor less than making plates of grained tin fit a plaster model. Hence I venture to bring before your notice a method which I have been using in my workroom for several months past, and which, whilst accomplishing all that the aforementioned swagers can do, is much quicker to work and can be purchased at merely a nominal price. You will readily see that it consists of a round gun-metal flask in two halves, with long lugs to guide the two parts into position. One half has an opening cut at one side into which a slot fits, which can be removed when one wants to line an impression. Each half has a nick cut on the inner surface to prevent the matrix twisting. The matrix used is compo; hence the name, "The Compo Swaging Flask." If you will allow me, I will pass round a few specimens of the work in its different stages for your inspection, and while they are going round I will put this paper aside and give a practical demonstration as to the working of this method.

### DIRECTIONS FOR WORKING THE COMPO SWAGING FLASK.

#### (*A*) Lining the Model.

(1) Remove the slot from the bottom of the flask, fill flask about three parts full of compo, and embed the impression tray in it, the handle of the same projecting through the opening. Cool under tap.

(2) Brush over with French chalk and water, or soap and water, and, having filled the top part of flask with compo (leaving it slightly higher in the centre), squeeze the two parts of the flask together in plaster press, or parallel vice, and when the two parts of flask are one-eighth of an inch apart, press thumb over slot to prevent any more compo coming out. Remove immediately, cool under tap, and part as soon as possible, then thoroughly chill.

(3) Cut a piece of lining metal of suitable shape, and mould on compo model with fingers, taking care to get all creases out, then partially squeeze up in vice, open, remove metal, trim, and get any creases out; squeeze up second time right home, open, place metal in position on impression, turn up outside edge at back slightly, so as to get better hold in the plaster, tap out compo setting, tray, etc., from flask and cast, remove compo, etc., with boiling water.

N.B.—The above instructions apply especially to edentulous cases. Should there be teeth standing, the holes in the impression must be lubricated and filled up with small portions of soft compo, which can be removed before casting.

Those practitioners who do not line their models should commence with paragraph *B*.

#### (*B*) Trial Plates.

(1) Put slot back in place, fill bottom part of flask with soft compo, press model into the same, not too deep, only just deep enough to get an impression. Press compo round sides of model until flat, cool under tap, and remove model, slightly bevel the edge of the impression obtained and lubricate.

(2) Fill top part of flask with compo, as before, squeeze both parts together in vice, cool at once under tap, part, and then thoroughly chill.

(3) Cut suitable piece of soft metal, mould it on to compo, partially squeeze up in vice, remove, trim, so that it goes over the gum margin about three-sixteenths of an inch, and squeeze up home. Take a smaller piece of metal, cut so as to reach gum margin only and one-eighth of an inch from palatine edge of No. 1, squeeze up on top of first plate, and then a third on top of the other two, also one-eighth of an inch shorter than No. 2. This is to give a sloping edge. Tack together with hard wax, and mount teeth, building up the gum margin with wax if required.

N.B.—Should teeth be standing, follow directions given in foot-note to paragraph 3 on Lining Plates.

### (*C*) Polishing Plate.

(1) Place trial plate, on which teeth have been mounted, on model and left forefinger round the incisor teeth, and squeeze onto the palate a piece of compo (shape of an egg) to get impression of lingual surface of plate and teeth. Hold model and compo under tap to cool same and also prevent teeth shifting.

(2) Fill bottom of flask three parts with compo and embed the under surface of the impression of the plate, cool, lubricate, fill top, and squeeze up as before. Cool at once, part, and thoroughly chill.

(3) Mould piece of soft metal, same as used for trial plates, onto impression, squeeze up partially, remove, trim, squeeze up home. Place on the trial plate, fix round the edges of the teeth with hot wax knife, having previously bent up slightly the corners at the back of the polisher so as to hold well in top plaster in flasking.

N.B.—When the polishing plate has been pulled off in the top plaster carefully polish the surface next to the vulcanite with whiting or French chalk so as to remove any graining that may be in the metal. If this precaution is taken the case comes out with a much better polish.

Any compo can be employed with this method, but to obtain the best results "swaging compo" must be used, especially prepared by Messrs. Ash & Sons, and the Dental Manufacturing Company. A flask takes one pound of compo, and should be lubricated before inserting the same. The lining metal should be very thin, as near the thickness of gold-leaf as possible. The soft metal for trial plates should be No. 6 gauge (Ash) and five and a half inches wide, for polishing plates, No. 4, three and one-quarter inches wide, to save waste.

Too much care cannot be taken to insure that the compo in each of the various stages is thoroughly hard before swaging the plates, particularly in warm weather. It is advisable for those who have not had previous experience with the "Compo Swaging Flask," to commence by swaging up trial and polishing plates, so as to get accustomed to this method before essaying to line models, the details of which might be found not quite so easy.—*Journal of the British Dental Association.*

